# Supplementary material for: Highly Efficient One-Pot Synthesis of Hexakis(m-phenyleneimine) Macrocyle Cm6 and the Thermostimulated Self-Healing Property through Dynamic Covalent Chemistry
Source: Polymers (Basel). 2023 Aug 25;15(17):3542. doi: 10.3390/polym15173542 (PMC10490300; doi:10.3390/polym15173542)
Supplement: Supplementary file 1 [file polymers-15-03542-s001.zip › polymers-2560995-supplementary.pdf]

Supplementary Materials

Highly efficient one-pot synthesis of hex-akis(m-phenyleneimine) macrocyle Cm6 and the thermostimu-lated self-healing property through dynamic covalent chemistry

Toshihiko Matsumoto

Department of Industrial Chemistry, Graduate School of Engineering, Tokyo Polytechnic University, Atsugi, Kanagawa 243-0297, Japan

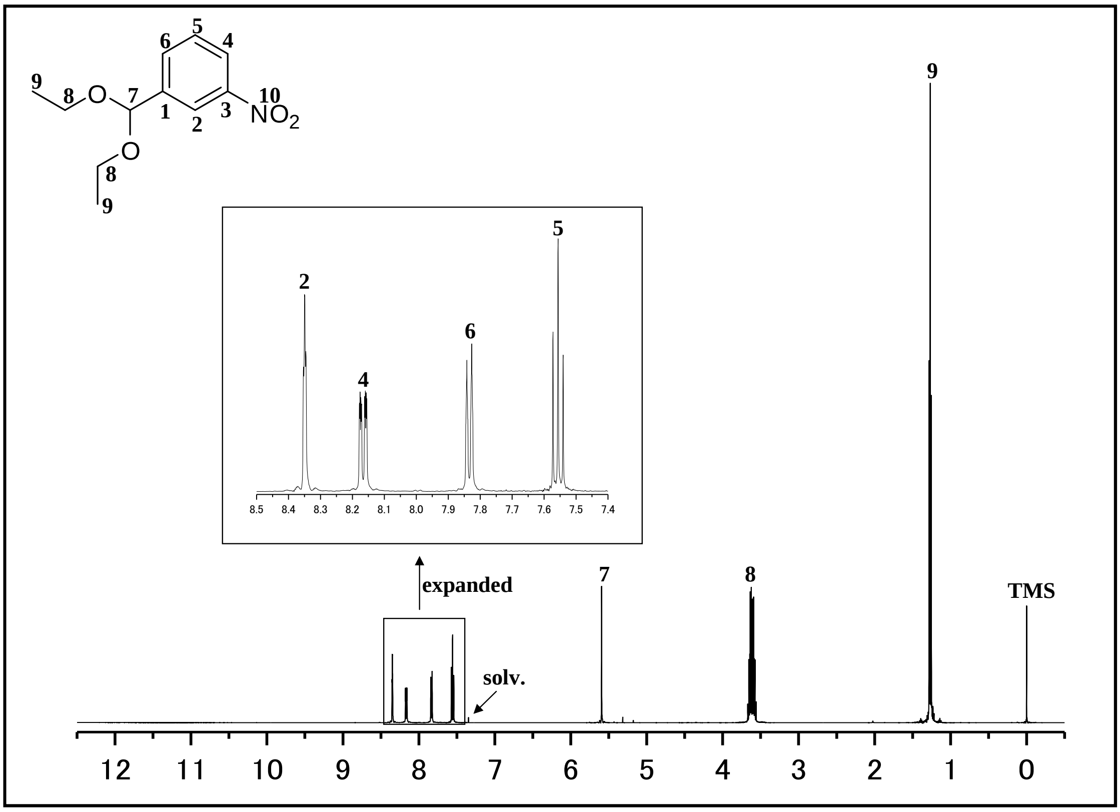

Figure S1. ^{1}H-NMR spectrum of *m*-nitrobenzaldehyde diethylacetal (**1**). (Solvent: $CDCl_3$, standard: tetramethylsilane).

^{1}H-NMR($CDCl_3$, δ); 1.27(*t*, $J_{9,8}$=7.0Hz, 6H, H-9), 3.56-3.67(*m*, 4H, H-8), 5.60(*s*, 1H, H-7), 7.56(*dd*, $J_{5,4}$=$J_{5,6}$=7.8Hz, 1H, H-5), 7.84(*d*, $J_{6,5}$=7,8Hz, 1H, H-6), 8.17(*d*, $J_{4,5}$=7.8Hz, 1H, H-4), 8.35(*s*, 1H, H-2).

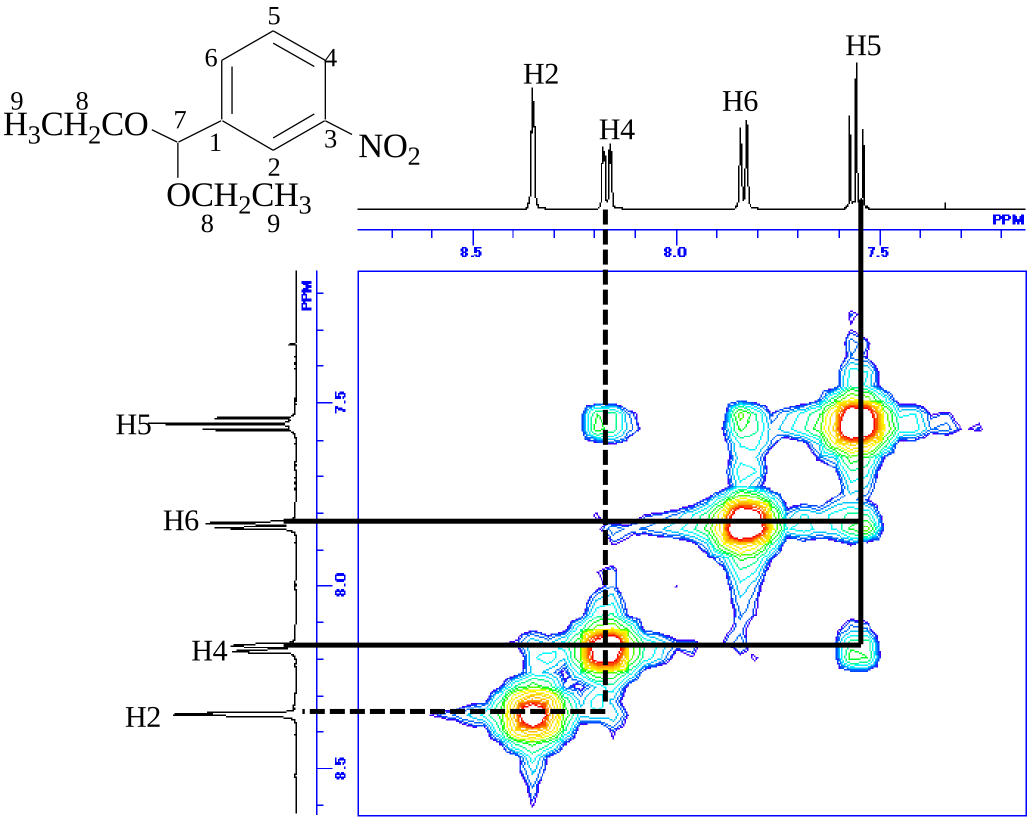

Figure S2. H,H-cosy spectrum of *m*-nitrobenzaldehyde diethylacetal (**1**). (Solvent: $CDCl_3$, standard: tetramethylsilane).

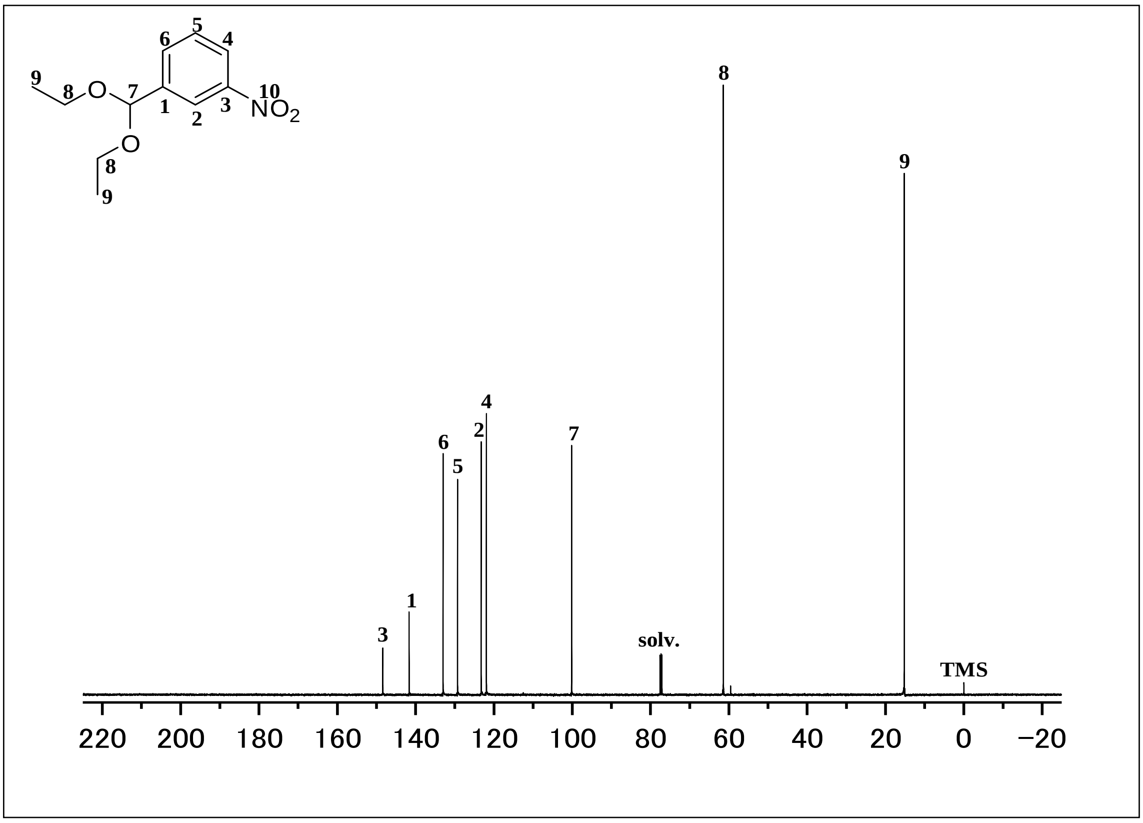

Figure S3. ^{13}C-NMR spectrum of *m*-nitrobenzaldehyde diethylacetal (**1**). (Solvent: $CDCl_3$, standard: tetramethylsilane).

^{13}C-NMR($CDCl_3$, δ); 15.20(C-9), 61.42(C-8), 100.16(C-7), 121.92(C-4), 123.26(C-2), 129.28(C-5), 132.98(C-6), 141.66(C-1), 148.38(C-3).

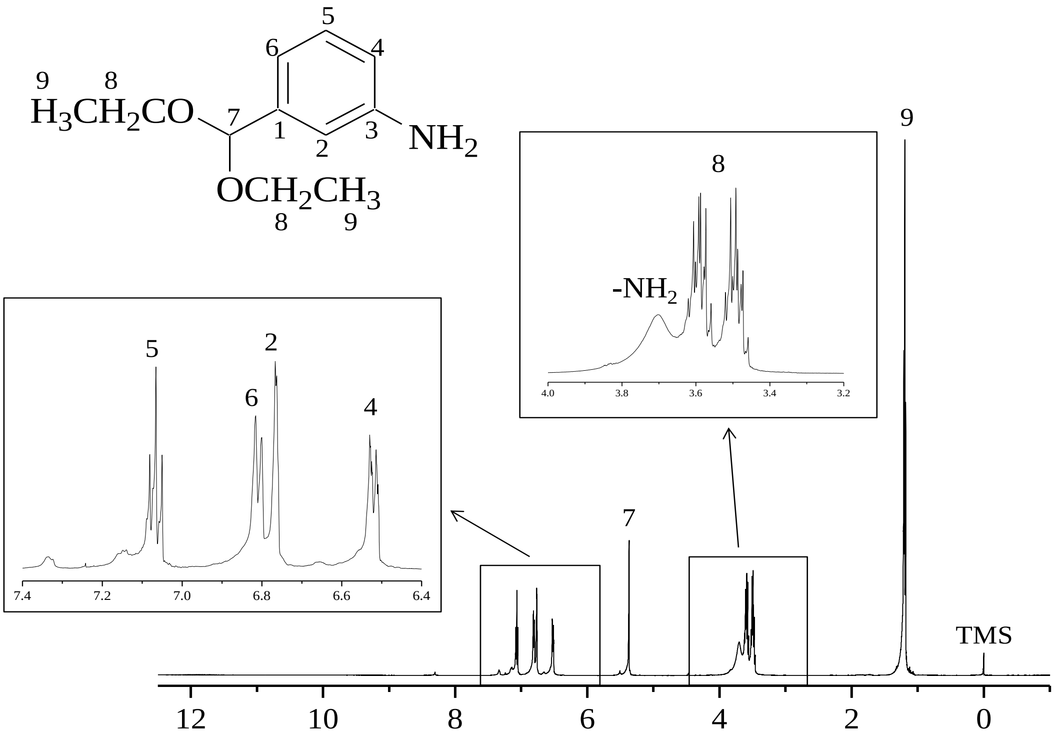

Figure S4. ^{1}H-NMR spectrum of *m*-aminobenzaldehyde diethylacetal (**2**). (Solvent: $CDCl_3$, standard: tetramethylsilane).

^{1}H-NMR($CDCl_3$, δ); 1.20(*t*, $J_{9,8}$=7.3Hz, 6H, H-9), 3.48-3.64(*m*, 4H, H-8), 3.70(*bs*, 2H, NH_2), 5.38(*s*, 1H, H-7), 6.57(*d*, $J_{4,5}$=7.9Hz, 1H, H-4), 6.78(*s*, 1H, H-7), 6.82(*d*, $J_{6,5}$=7.3Hz, 1H, H-6), 7.10(*dd*, $J_{5,4}$=$J_{5,6}$=7.9Hz, 1H, H-5).

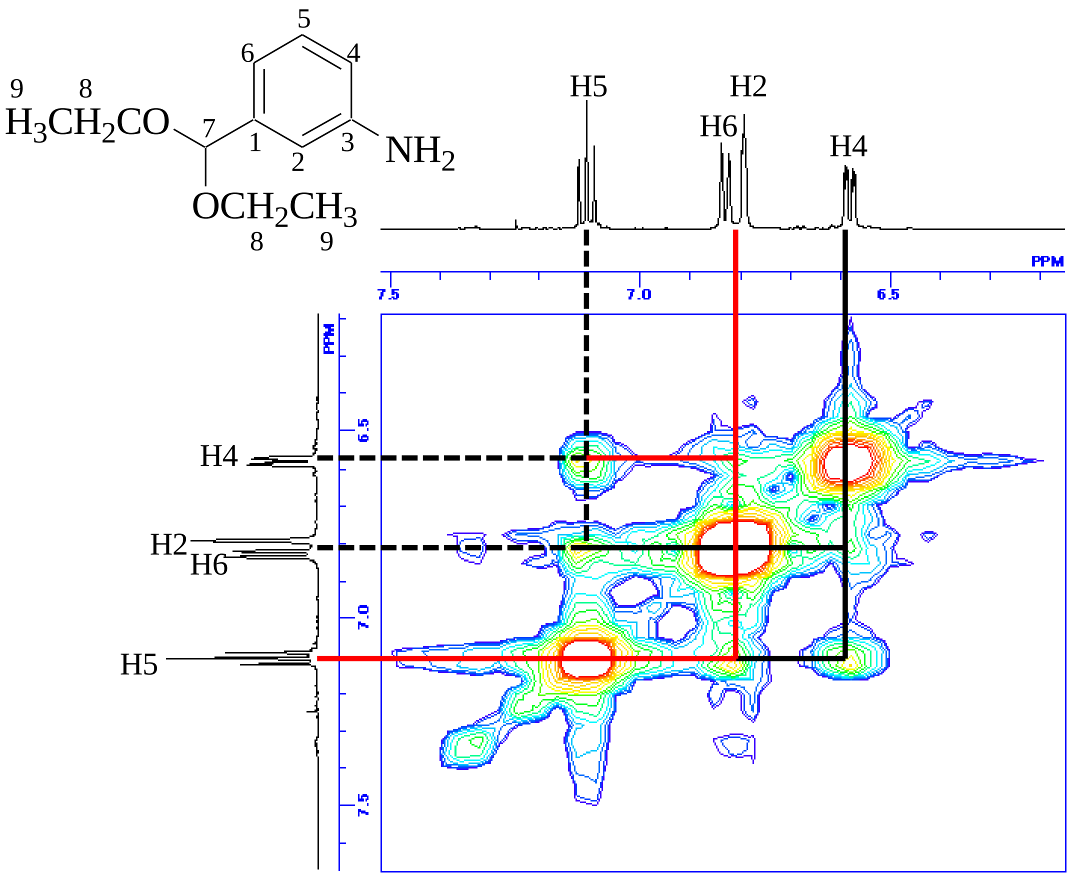

Figure S5. H,H-cosy spectrum of *m*-aminobenzaldehyde diethylacetal (**2**). (Solvent: CDCl3, standard: tetramethylsilane).

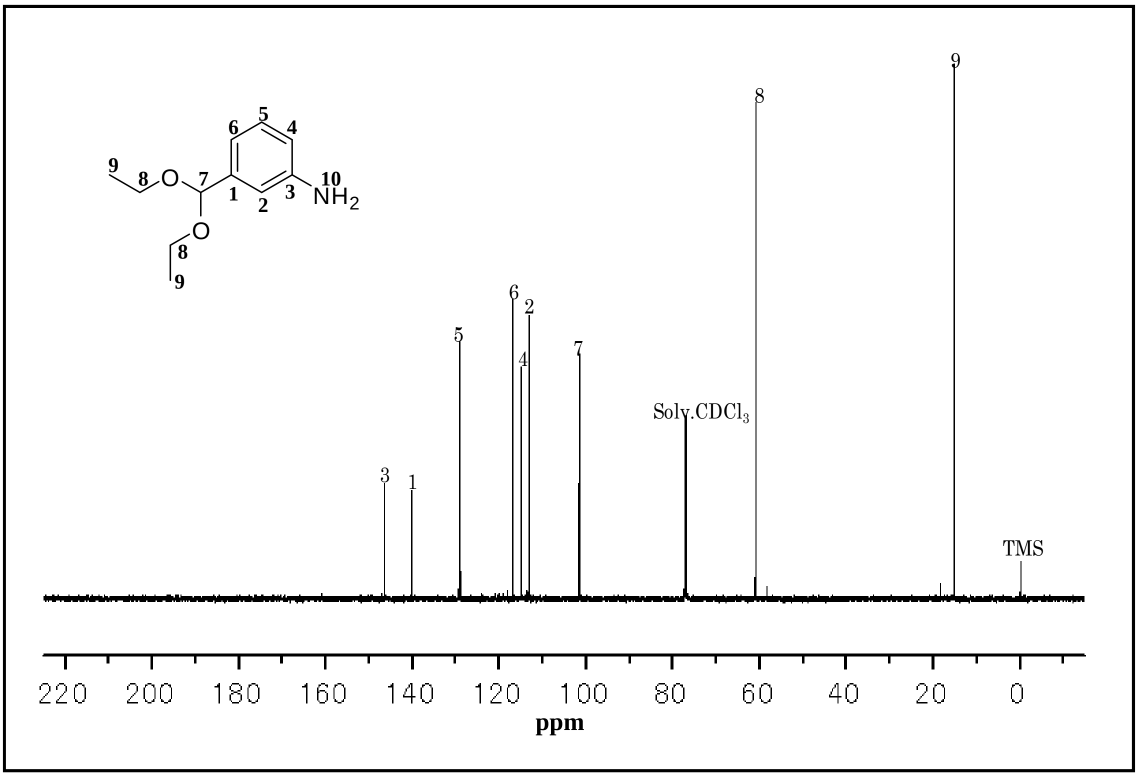

Figure S6. ^{13}C-NMR spectrum of *m*-aminobenzaldehyde diethylacetal (**2**). (Solvent: $CDCl_3$, standard: tetramethylsilane).

^{13}C-NMR($CDCl_3$, δ); 15.22(C-9), 61.01(C-8), 101.74(C-7), 113.23(C-2), 115.00(C-4), 116.64(C-6), 129.00(C-5), 140.26(C-1), 146.77(C-3).

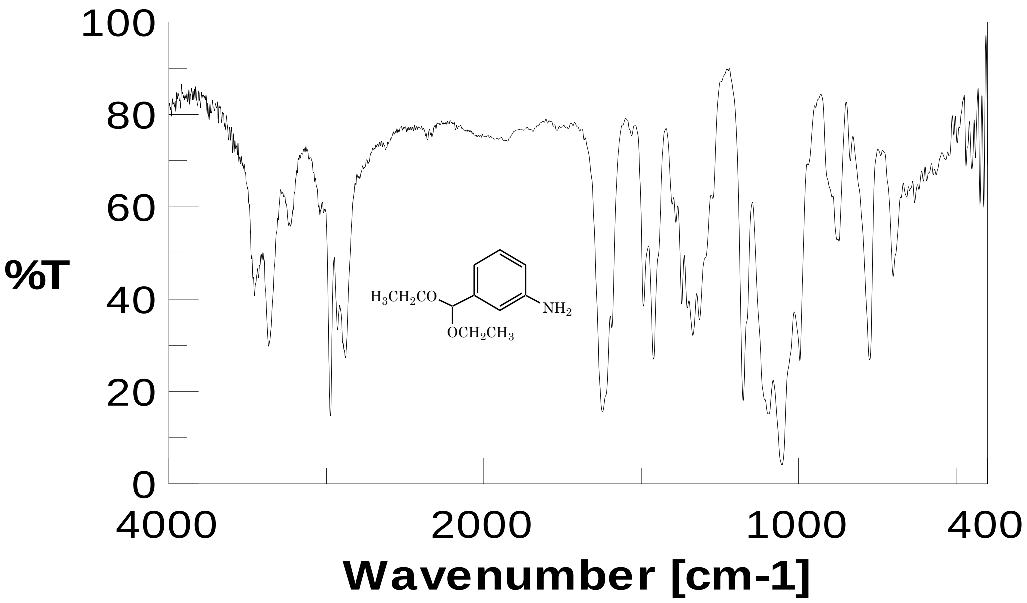

Figure S7. FT-IR spectrum of *m*-aminobenzaldehyde diethylacetal (**2**).

IR(liquid film, cm^{-1}); IR(liquid film, cm^{-1}); 3456 (NH_2 *as*), 3365(NH_2, sy), 2978~2883(acetal C-H), **c** 1623(aromatic amine), 1200 and 1113(acetal C-O-C *as* and *sy*).

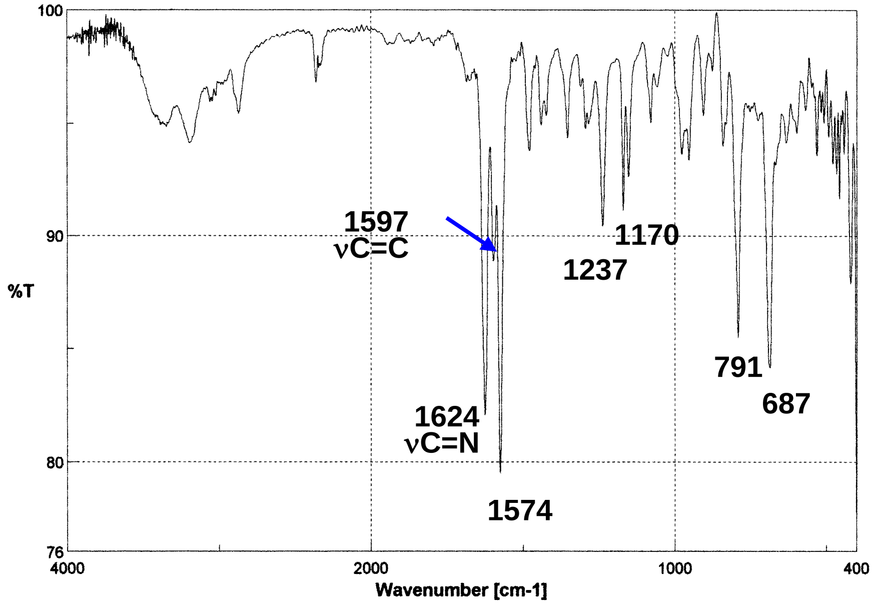

Figure S8. FT-IR spectrum of Cm6.

IR(KBr, cm^{-1}): 1624(νC=N), 1597(νC=C), 1574, 1237, 1170, 791, 687.

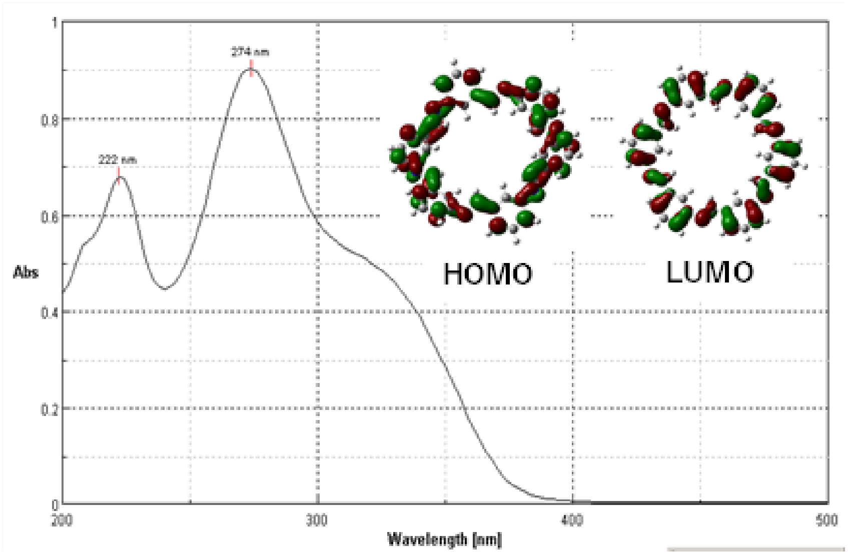

Figure S9. UV-Vis spectrum of Cm6 in THF and the frontier molecular orbitals, HOMO and LUMO obtained using Gaussian 03 (B3LYP, 6-31G(d,p)).

UV-vis (THF, nm): 330(sh), 274, 222.

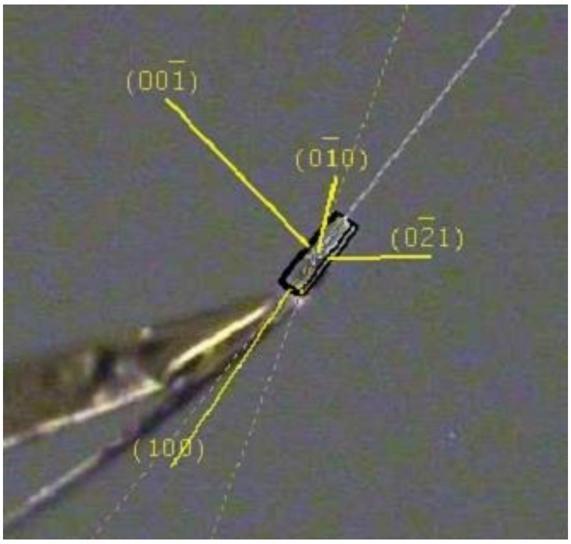

Figure S10. Microscopic image of single crystal of Cm6 mounted on glass fiber for X-ray analysis.

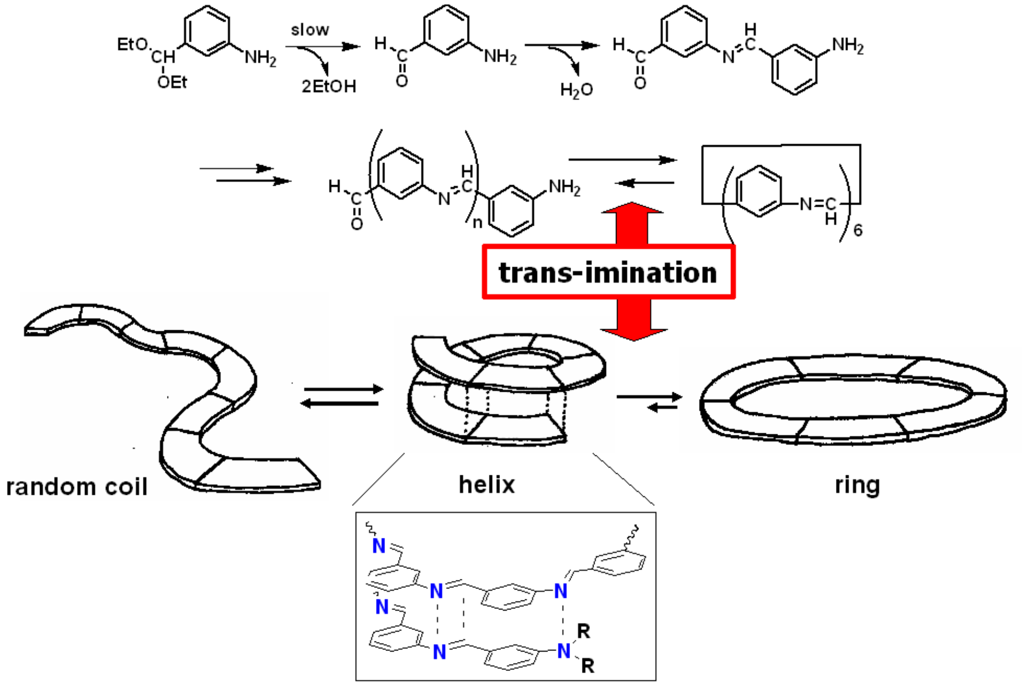

Figure S11. A possible mechanism for quantitative formation of macrocycle Cm6 through trans-imination (imine-metathesis) .

Table S1. Preparation of macrocycle Cm6 in various ratio of THF/H_2O (Solvent Effect)

entry	*m*-aminobenzaldehyde diethylacetal (g／mol)	THF (ml)	H_2O (ml)	conc. (M)	THF/ H_2O ratio(v/v), (mol/mol)	time[1] (day or h)	Yield (g／%)
1	0.50／0.026	1.9	0.1	1.28	19/1(4/1)	51d	0.10／38.5
2	0.50／0.026	1.75	0.25	1.28	7/1(3/2)	62d	0.08／30.9
3	0.50／0.026	1.55	0.45	1.28	7/2(3/4)	7d	0.18／69.2
4	0.50／0.026	1.25	0.75	1.28	5/3(1/3)	3d	0.10／38.5
5	0.50／0.026	1.0	1.0	1.28	1/1(1/3)	2d[2]	0.10／38.5
6	1.00／0.051	1.0	3.0	1.28	1/3(1/14)	22h[3]	0.22／86.2
7	0.50／0.026	0	2.0	1.28	0/1(0/1)	6d*2	0.40／76.9

1) time until precipitates appear 2) solidification during stirring 3) solidification next day

Table S2. Atomic coordinates (× 10^4) and equivalent isotropic displacement ($Å^2$ × 10^3) in the X-ray single crystal analysis of Cm6. U(eq) is defined as one third of the trace of the orthogonalized Uij tensor.

	x	y	z	U(eq)
N(1)	5870(3)	2601(6)	3469(6)	31(2)
N(2)	8480(3)	6139(6)	7258(6)	38(2)
N(3)	9670(2)	9930(6)	7521(6)	29(2)
N(4)	8770(3)	10206(6)	3995(6)	31(2)
N(5)	6110(2)	6668(6)	180(5)	29(2)
N(6)	4940(3)	2844(6)	-94(6)	37(2)
C(1)	5560(3)	3156(6)	4437(6)	21(2)
C(2)	4010(3)	2741(7)	4906(7)	24(2)
C(3)	3880(3)	3224(6)	5833(6)	21(2)
C(4)	5190(3)	4185(6)	6365(6)	19(2)
C(5)	6710(3)	4652(6)	5913(7)	24(2)
C(6)	6800(3)	4093(7)	4948(6)	24(2)
C(7)	8110(3)	5608(6)	6369(7)	25(2)
C(8)	9820(3)	7139(6)	7665(7)	25(2)
C(9)	11660(3)	7593(6)	8541(7)	23(2)
C(10)	12820(3)	8527(6)	8993(6)	21(2)
C(11)	12030(3)	9061(6)	8557(6)	20(2)
C(12)	10090(3)	8582(7)	7655(7)	23(2)
C(13)	8930(3)	7649(7)	7218(6)	23(2)
C(14)	9120(3)	9076(7)	7147(7)	25(2)
C(15)	8860(3)	10339(6)	6940(6)	20(2)
C(16)	7760(3)	11211(6)	7349(6)	21(2)
C(17)	7000(3)	11679(6)	6856(6)	17(2)
C(18)	7360(3)	11241(6)	5892(6)	18(2)
C(19)	8490(3)	10341(7)	5458(6)	26(2)
C(20)	9220(3)	9901(7)	5984(7)	24(2)
C(21)	8830(3)	9837(7)	4485(5)	28(2)
C(22)	8950(3)	9582(7)	2962(6)	24(2)
C(23)	10400(2)	10030(6)	2514(6)	20(2)
C(24)	10700(3)	9496(6)	1545(6)	18(2)
C(25)	9410(3)	8573(6)	1042(6)	19(2)
C(26)	7890(3)	8151(6)	1507(7)	23(2)
C(27)	7660(3)	8632(6)	2457(7)	23(2)
C(28)	6510(3)	7136(7)	1026(7)	27(2)
C(29)	4940(3)	5666(6)	-239(6)	21(2)
C(30)	2900(3)	5196(6)	-1136(6)	20(2)
C(31)	1710(3)	4238(6)	-156586)	19(2)
C(32)	2470(3)	3739(6)	-1151(6)	19(2)
C(33)	4500(3)	4177(7)	-250(6)	21(2)
C(34)	5570(3)	5160(6)	186(6)	24(2)
C(35)	5350(3)	3736(7)	273(7)	27(2)
C(36)	5830(3)	2441(6)	518(7)	25(2)
C(37)	6830(3)	1534(6)	66(7)	22(2)
C(38)	7530(3)	1111(6)	588(7)	22(2)
C(39)	7230(3)	1537(6)	1502(6)	21(2)
C(40)	6160(3)	2436(6)	1959(6)	21(2)
C(41)	5530(3)	2871(6)	1447(7)	25(2)
C(42)	5720(3)	2966(6)	2956(6)	24(2)
O(S1)	940(7)	6616(19)	2364(18)	131(8)*
O(S2)	3930(7)	8028(17)	3990(16)	123(7)*
O(S3)	3180(4)	7896(10)	5471(9)	46(3)*

(continued)

	x	y	z	U(eq)
O(S4)	2770(3)	6122(9)	5232(8)	47(3)*
O(S5)	1600(4)	4624(9)	3466(9)	49(3)*
O(S6)	1990(9)	5040(2)	2100(2)	154(10)*
O(S7)	2960(9)	6810(7)	3250(2)	164(7)*
C(S1)	2800(12)	5610(3)	3590(3)	143(8)*
C(S2)	3230(12)	6530(3)	4440(2)	141(7)*
C(S3)	2730(13)	7280(2)	4240(2)	148(8)*
C(S4)	2020(12)	5800(2)	2830(2)	154(8)*

*occupancy factor 0.5

Table S3. Bond lengths [Å] and angles [deg] in the X-ray single crystal analysis of Cm6.

N(1)-C(42)	1.260(11)
N(1)-C(1)	1.422(11)
N(2)-C(7)	1.279(12)
N(2)-C(8)	1.473(12)
N(3)-C(14)	1.223(12)
N(3)-C(15)	1.449(11)
N(4)-C(21)	1.231(11)
N(4)-C(22)	1.512(11)
N(5)-C(28)	1.211(11)
N(5)-C(29)	1.461(11)
N(6)-C(35)	1.281(12)
N(6)-C(36)	1.492(12)
C(1)-C(6)	1.371(14)
C(1)-C(2)	1.393(12)
C(2)-C(3)	1.343(12)
C(3)-C(4)	1.408(12)
C(4)-C(5)	1.428(12)
C(5)-C(7)	1.410(12)
C(5)-C(6)	1.404(12)
C(8)-C(13)	1.410(12)
C(8)-C(9)	1.355(14)
C(9)-C(10)	1.361(13)
C(10)-C(11)	1.421(11)
C(11)-C(12)	1.403(13)
C(12)-C(13)	1.360(13)
C(12)-C(14)	1.476(12)
C(15)-C(16)	1.370(13)
C(15)-C(20)	1.402(12)
C(16)-C(17)	1.403(12)
C(17)-C(18)	1.413(11)
C(18)-C(19)	1.414(13)
C(19)-C(20)	1.407(13)
C(19)-C(21)	1.425(12)
C(22)-C(23)	1.390(12)
C(22)-C(27)	1.391(13)
C(23)-C(24)	1.418(11)
C(24)-C(25)	1.355(11)
C(25)-C(26)	1.392(13)
C(26)-C(27)	1.383(13)
C(26)-C(28)	1.488(12)
C(29)-C(30)	1.414(12)
C(29)-C(34)	1.354(13)
C(30)-C(31)	1.398(12)
C(31)-C(32)	1.341(12)
C(32)-C(33)	1.417(13)
C(33)-C(34)	1.425(12)
C(33)-C(35)	1.413(12)
C(36)-C(41)	1.358(13)
C(36)-C(37)	1.404(13)
C(37)-C(38)	1.375(12)
C(38)-C(39)	1.338(13)
C(39)-C(40)	1.404(12)
C(40)-C(41)	1.372(12)
C(40)-C(42)	1.469(12)
	(continued)

C(S1)-C(S4)	1.449(10)
C(S1)-C(S2)	1.459(10)
C(S2)-C(S3)	1.449(10)
C(S3)-O(S7)	1.451(10)
O(S7)-C(S4)	1.452(10)
C(42)-N(1)-C(1)	119.5(8)
C(7)-N(2)-C(8)	119.1(9)
C(14)-N(3)-C(15)	117.2(9)
C(21)-N(4)-C(22)	117.2(8)
C(28)-N(5)-C(29)	117.5(8)
C(35)-N(6)-C(36)	118.4(9)
C(6)-C(1)-C(2)	117.6(8)
C(6)-C(1)-N(1)	122.7(8)
C(2)-C(1)-N(1)	119.7(8)
C(3)-C(2)-C(1)	121.7(9)
C(4)-C(3)-C(2)	121.1(8)
C(3)-C(4)-C(5)	119.5(8)
C(7)-C(5)-C(6)	119.3(8)
C(7)-C(5)-C(4)	124.8(8)
C(6)-C(5)-C(4)	115.9(8)
C(1)-C(6)-C(5)	124.2(8)
N(2)-C(7)-C(5)	123.2(9)
C(13)-C(8)-C(9)	119.6(9)
C(13)-C(8)-N(2)	122.2(9)
C(9)-C(8)-N(2)	117.8(9)
C(10)-C(9)-C(8)	121.9(9)
C(9)-C(10)-C(11)	120.0(9)
C(10)-C(11)-C(12)	117.3(8)
C(13)-C(12)-C(11)	121.8(8)
C(13)-C(12)-C(14)	116.8(9)
C(11)-C(12)-C(14)	121.3(9)
C(8)-C(13)-C(12)	119.3(8)
N(3)-C(14)-C(12)	121.7(9)
C(16)-C(15)-N(3)	118.4(8)
C(16)-C(15)-C(20)	118.0(8)
N(3)-C(15)-C(20)	123.5(8)
C(15)-C(16)-C(17)	123.0(8)
C(16)-C(17)-C(18)	119.2(8)
C(19)-C(18)-C(17)	118.6(8)
C(18)-C(19)-C(20)	119.9(8)
C(18)-C(19)-C(21)	122.4(9)
C(20)-C(19)-C(21)	117.7(9)
C(19)-C(20)-C(15)	121.2(8)
N(4)-C(21)-C(19)	122.5(9)
C(23)-C(22)-C(27)	120.3(9)
C(23)-C(22)-N(4)	115.3(8)
C(27)-C(22)-N(4)	124.3(8)
C(22)-C(23)-C(24)	119.1(8)
C(23)-C(24)-C(25)	121.2(8)
C(26)-C(25)-C(24)	118.3(8)
C(27)-C(26)-C(25)	122.7(8)
C(27)-C(26)-C(28)	114.7(8)
C(25)-C(26)-C(28)	122.5(9)
C(26)-C(27)-C(22)	118.3(8)
N(5)-C(28)-C(26)	120.7(8)

(continued)

C(30)-C(29)-C(34)	117.4(8)
C(30)-C(29)-N(5)	118.0(8)
C(34)-C(29)-N(5)	124.5(8)
C(29)-C(30)-C(31)	120.2(9)
C(32)-C(31)-C(30)	121.7(8)
C(31)-C(32)-C(33)	120.4(8)
C(34)-C(33)-C(32)	116.7(8)
C(34)-C(33)-C(35)	117.6(9)
C(32)-C(33)-C(35)	125.6(9)
C(33)-C(34)-C(29)	123.5(9)
N(6)-C(35)-C(33)	122.2(9)
C(41)-C(36)-C(37)	119.8(9)
C(41)-C(36)-N(6)	124.3(8)
C(37)-C(36)-N(6)	115.8(9)
C(38)-C(37)-C(36)	118.4(9)
C(39)-C(38)-C(37)	121.6(9)
C(38)-C(39)-C(40)	120.5(8)
C(41)-C(40)-C(39)	118.2(8)
C(41)-C(40)-C(42)	116.6(8)
C(39)-C(40)-C(42)	125.2(8)
C(36)-C(41)-C(40)	121.4(8)
N(1)-C(42)-C(40)	121.9(8)
C(S4)-C(S1)-C(S2)	105(3)
C(S3)-C(S2)-C(S1)	113(3)
C(S2)-C(S3)-O(S7)	100(2)
C(S3)-O(S7)-C(S4)	111(3)
C(S1)-C(S4)-O(S7)	105(3)

Table S4. Anisotropic displacement parameters ($Å^2 \times 10^3$) in the X-ray single crystal analysis of Cm6. The anisotropic displacement factor exponent takes the form: -2 pi^2 [h^2 a^{*2} U11 + … + 2 h k a* b* U12]

	U11	U22	U33	U23	U13	U12
N(1)	33(4)	28(4)	29(4)	14(3)	-3(3)	-4(3)
N(2)	34(5)	23(4)	38(4)	2(3)	1(3)	1(3)
N(3)	35(4)	30(4)	26(4)	17(3)	5(3)	4(3)
N(4)	38(4)	22(3)	30(4)	10(3)	7(3)	1(3)
N(5)	38(4)	33(4)	29(4)	25(3)	3(3)	4(3)
N(6)	39(5)	24(4)	35(4)	6(3)	6(4)	4(3)
C(1)	20(4)	26(4)	20(4)	15(3)	-1(3)	1(3)
C(2)	21(4)	23(4)	27(4)	13(3)	0(3)	1(3)
C(3)	20(4)	18(4)	29(4)	17(3)	1(3)	-2(3)
C(4)	24(4)	15(3)	18(4)	9(3)	-1(3)	0(3)
C(5)	21(4)	20(3)	30(4)	14(3)	0(3)	-4(3)
C(6)	24(4)	36(4)	20(4)	20(3)	4(3)	5(3)
C(7)	34(5)	18(3)	21(4)	9(3)	7(3)	-1(3)
C(8)	24(4)	20(4)	28(4)	10(3)	7(3)	5(3)
C(9)	24(4)	19(4)	29(4)	15(3)	6(3)	3(3)
C(10)	20(4)	27(4)	19(4)	15(3)	3(3)	0(3)
C(11)	22(4)	16(3)	24(4)	11(3)	6(3)	4(3)
C(12)	24(4)	31(4)	25(4)	21(3)	6(3)	8(3)
C(13)	29(4)	31(4)	8(3)	8(3)	1(3)	10(3)
C(14)	34(5)	24(4)	23(4)	19(3)	-3(3)	-5(3)
C(15)	17(4)	21(3)	25(3)	14(3)	2(3)	-2(3)
C(16)	22(4)	24(4)	15(3)	10(3)	-1(3)	-1(3)
C(17)	18(4)	16(3)	14(3)	4(3)	7(3)	4(3)
C(18)	14(4)	25(4)	21(4)	16(3)	6(3)	-1(3)
C(19)	19(4)	34(4)	21(4)	10(3)	7(3)	0(3)
C(20)	20(4)	21(4)	28(4)	11(3)	2(3)	4(3)
C(21)	42(5)	21(4)	17(4)	5(3)	10(4)	13(4)
C(22)	19(4)	32(4)	21(4)	15(3)	2(3)	2(3)
C(23)	24(4)	17(3)	17(4)	7(3)	-3(3)	-2(3)
C(24)	20(4)	21(3)	16(3)	11(3)	3(3)	3(3)
C(25)	17(4)	20(4)	18(4)	8(3)	3(3)	6(3)
C(26)	17(4)	13(3)	38(4)	13(3)	-1(3)	2(3)
C(27)	24(4)	20(3)	33(4)	21(3)	1(3)	-3(3)
C(28)	39(5)	22(4)	23(4)	13(3)	10(4)	-2(4)
C(29)	17(4)	20(3)	16(3)	1(3)	2(3)	2(3)
C(30)	24(4)	19(4)	14(3)	5(3)	1(3)	5(3)
C(31)	20(4)	18(3)	9(3)	0(3)	-3(3)	2(3)
C(32)	17(4)	17(3)	21(4)	8(3)	4(3)	3(3)
C(33)	16(4)	28(4)	21(3)	13(3)	3(3)	4(3)
C(34)	20(4)	22(4)	19(4)	2(3)	0(3)	-6(3)
C(35)	36(5)	16(3)	20(4)	5(3)	-6(3)	-2(3)
C(36)	22(4)	17(3)	37(4)	15(3)	3(3)	-1(3)
C(37)	26(4)	14(3)	24(4)	7(3)	3(3)	0(3)
C(38)	23(4)	9(3)	29(4)	6(3)	-5(3)	-2(3)
C(39)	26(4)	15(3)	2084)	8(3)	-5(3)	1(3)
C(40)	20(4)	13(3)	14(3)	-3(3)	-2(3)	-3(3)
C(41)	23(4)	6(3)	38(4)	6(3)	3(3)	-4(3)
C(42)	38(4)	14(3)	20(4)	8(3)	-3(3)	6(3)

Table S5. Hydrogen coordinates (× 10^4) and isotropic displacement parameters (Å^2 × 10^3) in the X-ray single crystal analysis of Cm6.

	x	y	z	U(eq)
H(2)	3046	2112	4569	29
H(3)	2907	2920	6128	25
H(4)	5072	4513	7005	23
H(6)	7776	4377	4634	29
H(7)	8810	5864	6006	30
H(9)	12138	7255	8841	27
H(10)	14136	8816	9587	25
H(11)	12778	9702	8857	24
H(13)	7560	7350	6630	28
H(14)	8069	8730	6526	30
H(16)	7508	11505	7983	25
H(17)	6264	12272	7160	21
H(18)	6860	11538	5551	22
H(20)	9959	9309	5693	28
H(21)	9101	9205	4209	33
H(23)	11162	10671	2844	24
H(24)	11799	9785	1250	22
H(25)	9546	8230	403	22
H(27)	6668	8329	2751	27
H(28)	5942	6858	1379	33
H(30)	2346	5525	-1441	24
H(31)	350	3940	-2154	22
H(32)	1661	3101	-1456	23
H(34)	6760	5471	794	29
H(35)	6237	4105	905	32
H(37)	7010	1228	-572	27
H(38)	8235	515	298	27
H(39)	7725	1235	1839	26
H(41)	4880	3473	1743	30
H(42)	5324	3589	3216	29
